# *Camellia japonica* Essential Oil Inhibits *α*-MSH-Induced Melanin Production and Tyrosinase Activity in B16F10 Melanoma Cells

**DOI:** 10.1155/2021/6328767

**Published:** 2021-11-16

**Authors:** Si Young Ha, Ji Young Jung, Jae-Kyung Yang

**Affiliations:** Department of Environmental Materials Science/Institute of Agriculture and Life Science, Gyeongsang National University, Jinju 52828, Republic of Korea

## Abstract

Essential oils are aromatic oils extracted from the leaves, stems, peels, petals, and roots of aromatic plants grown in nature or grown in organic methods and have various medical effects as natural substances. The essential oil extracted from *Camellia japonica* seeds exhibits various functional properties; however, its tyrosinase inhibitory activity has not been investigated extensively. This study is performed to investigate the chemical composition and tyrosinase inhibitory activity of *Camellia japonica* seed essential oil (CJS-EO). Hexamethylcyclotrisiloxane (42.36%) and octamethylcyclotetrasiloxane (23.28%) are the two primary components of CJS-EO, as identified via gas chromatography-mass spectrometry. The inhibitory activities of CJS-EO and positive control arbutin are further evaluated against mushroom tyrosinase. The results show that CJS-EO and arbutin inhibit tyrosinase activity. Moreover, CJS-EO significantly inhibits melanogenesis in the *α*-melanocyte-stimulating hormone-treated group, and a significant amount of melanin is suppressed. To ascertain the cause of the CJS-EO tyrosinase inhibitory effect and melanin reduction effect, genetic and protein analyses are performed. Based on our results, we tentatively conclude that CJS-EO can inhibit melanocytes from harmful factors such as tyrosinase-related protein. These results demonstrate that CJS-EO possesses potent antityrosinase activity and may be a good skin-whitening agent.

## 1. Introduction

Skin color is affected by pigments such as melanin in the *epidermis*, hemoglobin in the blood vessels of the dermis, and carotene in the subcutaneous tissue. Among them, the external skin color is determined by the amount and distribution of melanin pigment [1]. Enzymes involved in melanin synthesis are well known as tyrosinase, tyrosinase-related protein-1 (TRP-1), and dopachrome tautomerase (DCT, TRP-2) [2]. Among them, tyrosinase is an enzyme acting in the initial reaction, which is the rate-determining step of melanin synthesis, and oxidizes tyrosinase to DOPA-quinone [3]. Therefore, substances that inhibit tyrosinase, TRP-1, and TRP-2 can inhibit melanin synthesis and thus have a skin-whitening function [4]. Melanin plays an important role in protecting the skin from UV rays and external harmful factors in human skin. However, when it is produced in excess and accumulated on the skin, it can cause melasma, freckles, skin spots, etc. and can lead to cell death due to the toxicity of melanin precursors and diseases such as skin cancer [5–7].

L-ascorbic acid, arbutin, and lactic acid have been developed as representative melanin production inhibitors, but their usage is strictly regulated due to skin irritation or safety issues. Therefore, research is being actively conducted to find a natural whitening agent that is safe and effective [8].

Plant essential oils are known to exhibit various functional properties, such as strong antibacterial activity and antiaging and skin regeneration effects [9, 10]. *Cinnamomum cassia* essential oil [4], *Polygonum odoratum* essential oil [11], and *Vitex negundo* Linn leaf essential oil [12] have been reported to possess tyrosinase inhibitory activities.

*Camellia japonica* (Japanese name of “tsubaki”) is a popular tree as both a garden plant and a source of oil material and folk medicine in Japan [13]. In Korea, *C. japonica* is an evergreen tree belonging to the family Theaceae and the genus *Camellia* [14]. *C. japonica* seed oil has a long history of use as a cosmetic protectant to ensure skin and hair health and as a soothing agent [15]. *C. japonica* seed oil has been reported to exhibit various biological activities, including antioxidant activity [16], antibacterial activity [17], anti-inflammatory activity [18], and skin barrier function [19]. Despite its widespread use, few studies have examined the effects of *C. japonica* seed oil on skin-whitening-associated tyrosinase activity.

Therefore, in this study, the chemical composition of *C. japonica* seed essential oil (CJS-EO) was investigated using gas chromatography-mass spectrometry (GC-MS), and the tyrosinase inhibitory activity of this essential oil was examined. To address this inhibitory activity, the effects of CJS-EO on *α*-MSH-stimulated melanogenesis and tyrosinase inhibition in B16F10 melanoma cells were assessed.

## 2. Materials and Methods

### 2.1. Plant Materials

*C. japonica* seeds were purchased in March 2021 from the Seohyeon Herbal Medicine Farming Association, Nonsan, South Korea. Plant materials were identified by Hee-Gon Kang, a representative from the experimental forest of Gyeongsang National University.

### 2.2. Reagents

Mushroom tyrosinase was purchased from T-3824 (Sigma–Aldrich, USA), and mouse B16F10 melanoma was purchased from CRL-6475 (American Type Culture Collection, Manassas, VA, USA). Media and reagents required for cell culture were purchased from Invitrogen (USA), Sigma (USA), and Nunc (USA). For western blotting, a western blot kit and a semidry transfer system (Bio-Rad, USA) were used, and the results were confirmed using an image analysis system (Bio-Rad, USA). Antibodies were purchased from Santa Cruz Biotech (USA). Dimethyl sulfoxide (DMSO) was purchased from Sigma–Aldrich (St. Louis, MO, USA).

### 2.3. Extraction and Sample Preparation

Extraction was performed using a method published previously with slight modifications [4]. Briefly, *C. japonica* seeds (500 g) were placed in a vessel and extracted via distillation using 2000 mL of water for 4 h. The vapor generated was cooled using a closed cooling system, and the resulting liquid was collected in a container. The oil floated toward the top of the distilled liquid, whereas the water settled into the lower phase of the liquid; hence, CJS-EO was obtained by removing the upper phase of the liquid, which contained the desired oil, and then stored at −20°C until use. A 500 ppm stock solution of CJS-EO in DMSO was used for the cell experiments.

### 2.4. GC-MS Analysis

The volatile components of CJS-EO were analyzed using GC-MS (Clarus 600 GC-MS, Perkin Elmer, Shelton, CT, USA). The analysis column used was PerkinElmer Elite-5 ms (30 mm × 0.3 mm × 0.25 *μ*m). Helium gas (1.0 mL/min) was used as the mobile phase. The oven temperature was increased from 40°C to 100°C at a rate of 10°C/min and then maintained for 1.0 min. Next, the temperature was increased to 230°C at a rate of 10°C/min and then maintained for 5 min. The temperature of the injector was set to 200°C, and the temperature of the detector was set to 250°C. The analyzed results were identified using the NIST Mass Spectral Search Program (Version 2.0 g, National Institute of Standards and Technology, Gaithersburg, MD, USA).

### 2.5. Cell Culture

B16F10 mouse melanoma cells were purchased from the Korea Cell Line Bank. Cell culture was performed using DMEM (WELGENE, Daegu, Korea) supplemented with 10% FBS (fetal bovine serum, WELGENE, Daegu, Korea) and 1% penicillin-streptomycin (Gibco BRL) at 37°C and 5% CO_2_ conditions. In order to solve the overdensity phenomenon caused by the proliferation of cell numbers, cultured B16F10 cells were maintained at an appropriate number using trypsin (Hyclone, USA).

### 2.6. Cell Viability Assay

B16F10 melanoma cells were seeded at 3 × 10^4^/mL in a 6-well plate for cell culture. CJS-EO was treated at an appropriate concentration (31.25 ppm to 500 ppm) per well. The medium was removed after 72 hours, and 200 *μ*L 3-(4,5-Dimethylthiazol-2-yl)-2,5-Diphenyltetrazolium bromide (MTT) solution (Promega, Madison, WI, USA) was treated. MTT reagent was cleanly removed after incubation in a 37°C CO_2_ incubator for 2 hours. For the stained cells, 2 mL of DMSO was treated to dissolve all of the formazan generated in the wells. Cell viability was measured by absorbance at 540 nm with an ELISA reader (SpectraMax 190, Molecular Devices LLC, San Jose, CA, USA).

### 2.7. Tyrosinase Activity Assay

Tyrosinase activity was estimated by measuring the rate of L-DOPA oxidation [20]. B16F10 cells (2 × 10^5^ cells/well) were treated with *α*-melanocyte stimulating hormone (*α*-MSH) and CJS-EO and cultured for 3 days. Cells were harvested, lysis buffer (1% Triton X-100, 0.1 M PMSF) was added, and then the cells were lysed by reacting at 4 °C for 1 hour. The supernatant was collected by centrifugation at 12,000×g for 15 minutes. After quantification of protein in the collected supernatant, 10 mmol/mL L-DOPA was added. Next, cells were incubated in a CO_2_ incubator at 37°C for 30 min and the absorbance at 490 nm was recorded using an absorbance microplate reader (SpectraMax 190, Molecular Devices LLC, San Jose, CA, USA). The data obtained were calculated using the following formula: tyrosinase activity (%) = (OD 490 of sample/OD 490 of control) ×100.

### 2.8. Melanin Content Assay

B16F10 cells were seeded in a six-well plate (2 × 10^5^ cells/well) for 12 h after renewing the culture medium of the cells treated with different concentrations of CJS-EO and *α*-MSH for 48 h and washing with PBS twice [21]. B16F10 cells were treated with *α*-MSH and CJS-EO together, cultured for 3 days, and the cells were harvested and washed twice with phosphate-buffered saline (PBS). After that, it was treated with 1N NaOH containing 10% DMSO and reacted at 80°C for 1 hour. For melanin content analysis, the absorbance was measured at 475 nm using an absorbance microplate reader (SpectraMax 190, Molecular Devices LLC, San Jose, CA, USA). The percentage value of the CJS-EO-treated cells was calculated with respect to the negative control. In detail, BCA Protein Assay Kit (Thermo Fisher Scientific, Waltham, US) was used to determine the protein concentration of each sample. The melanin content was normalized to the cellular protein concentration (absorbance melanin/*μ*g protein).

### 2.9. Investigation of Genes

B16F10 melanoma cells were inoculated in a 100 mm dish at a density of 1 × 10^6^ cells in DMEM (GIBCO, USA) supplemented with 10% bovine serum and 1% antibiotics; subsequently, they were incubated in 5% CO_2_ at 37°C. After changing to a fresh 10% DMEM medium, CJS-EO was added to a culture plate and cultured for 3 d, and 1% Amisoft as a surfactant was added in an amount corresponding to 1/1,000 of the medium volume. After 3 d, 1 mL of TRIzol (Invitrogen, USA) was added to the cells to investigate the mRNA expression level of whitening-related genes, and the RNA was isolated using Invitrogen's RNA isolation method. After quantifying the amount of RNA at 260 nm using an ultraviolet detector, reverse transcription-polymerase chain reaction (RT-PCR) was performed. For the RT-PCR, an all-in-one RT-PCR kit (Super Bio, Korea) was used, the experiment was performed based on the manufacturer's instructions, and the primers and reaction conditions were as follows: the sequence of actin was 5′-GAG ACC TTC AAC ACC CCA GCC-3'; antisense, 5′-GGC CAT CTC TTG CTC GAA GTC-3'; reverse transcription at 50°C for 30 min; reverse transcriptase inactivated at 96°C for 3 min, 94°C for 30 s, and 62°C for 1 min, followed by 25 cycles at 72°C for 1 min. The sequence of tyrosinase was 5′-GGC CAG CTT TCA GGC AGA GGT-3'; antisense, 5′-TGG TGC TTC ATG GGC AAA ATC-3'; denatured at 90°C for 30 s; reverse transcription at 60°C for 30 min; reverse transcriptase inactivated at 94°C for 1 min. Thereafter, a PCR was performed for 30 cycles at 94°C for 30 s, 56°C for 30 s, and 72°C for 1 min. The sequence of tyrosinase-related protein 1 (TRP-1) was 5′-GCT GCA GGA GCC TTC TTT CTC-3'; antisense, 5′-AAG ACG CTG CAC TGC TGG TCT-3'; denatured at 90°C for 30 s; reverse transcription at 60°C for 30 min; reverse transcriptase inactivated at 94°C for 1 min. Thereafter, a PCR was performed for 30 cycles at 94°C for 30 s, 56°C for 30 s, and 72°C for 1 min. The sequence of tyrosinase-related protein 2 was 5′-TGA CCG TGA GCA ATG GCC-3'; antisense, 5′-CGG TTG TGA CCA ATG GGT GCC-3'; reverse transcription at 50°C for 30 min; inactivation of reverse transcriptase at 96°C for 3 min, 94°C for 1 min, and 60°C for 1 min. Subsequently, 72 PCR reactions were performed in 25 cycles for 1 min.

### 2.10. Investigation of Protein Expression

Mouse B16F10 melanoma cells were inoculated in a 100 mm dish at a density of 5 × 10^5^ cells in DMEM supplemented with 10% FBS and 1% antibiotics and then cultured in 5% CO_2_ at 37°C for 1 d. Subsequently, they were exchanged with a new medium, and CJS-EO was treated at different concentrations and cultured for 3 d. A 1% aqueous solution of Amisoft as a surfactant was added at a volume corresponding to 1/1000 of the medium volume. The cultured cells were washed with PBS and transferred to a 1.5 mL microtube and then to a cell disruption buffer (40 mM Tris-Cl [pH 7.4], 10 mM EDTA, 120 mM NaCl, 0.1% NP-40, 1 mM PMSF, and protease inhibitor cocktail). After the cells were destroyed by addition, centrifugation was performed at 15,000 rpm at 4°C for 10 min, and the supernatant was recovered to separate the proteins. The isolated proteins were quantified using Sigma's BCA method, and SDS-PAGE was performed. After transferring the SDS-PAGE gel to the PVDF membrane, the protein was labeled using a secondary antibody conjugated with a primary antibody and peroxidase and then exposed to an X-ray film using a western blot detection kit (Intron, Korea). Subsequently, expression levels were analyzed.

## 3. Results

### 3.1. Chemical Composition of CJS-EO

CJS-EO was obtained at a yield of 18% w/w. The chemical composition of CJS-EO was analyzed using GC-MS (Table 1). The peaks were separated in GC, and 17 compounds were identified via MS, which constituted 90% of the total peak area (Table 1). The main chemical components of the CJS-EO were hexamethylcyclotrisiloxane (42.36%), octamethylcyclotetrasiloxane (23.28%), decamethylcyclopentasiloxane (5.81%), hexanedioic acid (5.56%), and vanillin (2.96%). In previous research, GC-MS analysis detected hexamethylcyclotrisiloxane in leaf and stem extracts of *Bauhinia acuminata* Linn [22]. The volatile components in *Moringa oleifera* were mainly composed of esters, acids, aldehydes, and hydrocarbons, and hydrocarbons are mainly hexamethylcyclotrisiloxane [23]. Similarly, in our study, hexamethylcyclotrisiloxane was confirmed as the main chemical component of CJS-EO. One of the main chemical components of CJS-EO, vanillin, was detected in the essential oil of *Eugenia caryophyllata* and *Ocimum basilicum* [24], *Hyssopus officinalis L*. (Lamiaceae) essential oil [25], and *Calea clematidea* essential oil [26]. In previous studies, the inhibitory effect of vanillin on the activities of monophenolase and diphenolase contained in tyrosinase was previously reported [27]. Interestingly, low molecular weight cyclic volatile methylsiloxane compounds including hexamethylcyclotrisiloxane have been used in a variety of cosmetics and personal care products and many other consumer products [28]. In future, the compound responsible for inhibiting melanin production and tyrosinase activity is to be extracted and could be used in natural cosmetics or medicine.

### 3.2. Cell Viability of Melanoma Cells Induced by CJS-EO

**Figure 1:**
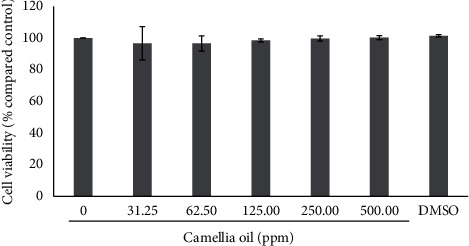
The effects of *C. japonica* seed essential oil (CJS-EO) on the cell viability of *α*-MSH-treated B16 cells. The data are representative of three independent experiments and expressed as the mean ± standard error of the mean (SEM).

Our results showed that murine melanoma B16F10 cells treated with a concentration of 31.25 to 500 ppm of CJS-EO for 24 h did not induce any changes in cell viability. At a concentration of 31.25 to 500 ppm, the cell viability was 90% to 100%, which indicated low cytotoxicity (Figure 1). Therefore, all concentrations (31.25 to 500 ppm) were suitable for further evaluating the effects of CJS-EO on tyrosinase activity and melanin synthesis in B16F10 cells.

### 3.3. Inhibition of Intracellular Tyrosinase Activity of CJS-EO

**Figure 2:**
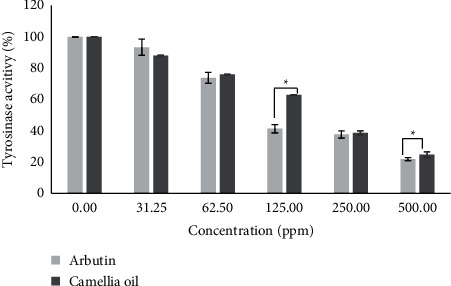
The tyrosinase inhibitory activities of *C. japonica* seed essential oil (CJS-EO). The data are representative of three independent experiments and expressed as the mean ± standard error of the mean (SEM). ^*∗*^*p* < 0.05 compared with arbutin each at the same concentration.

The effect of CJS-EO on the oxidation of L-DOPA catalyzed by tyrosinase, as well as that of arbutin, a well-known tyrosinase inhibitor, was investigated. As shown in Figure 2, CJS-EO and arbutin exhibited potent inhibitory effects on L-DOPA oxidase activity in a dose-dependent manner. The results show that CJS-EO and arbutin exhibited similar tyrosinase inhibitory activities. Based on statistical analysis, arbutin had a significantly higher tyrosinase inhibitory activity than CJS-EO at 125 and 500 ppm. However, at 31.25, 32.50, and 250 ppm, similar inhibitory effects (not significant) were indicated on tyrosinase when compared with the tyrosinase inhibitory activity of CJS-EO on the well-known tyrosinase inhibitor arbutin.

### 3.4. Effect of CJS-EO on Melanin Content in B16F10 Cells

**Figure 3:**
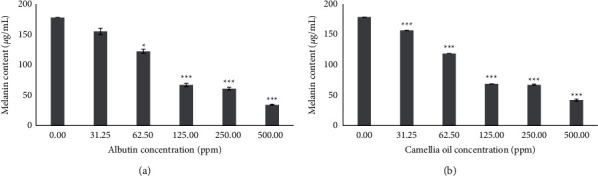
The effects of arbutin (a) and *C. japonica* seed essential oil (CJS-EO) (b) on melanin content in *α*-MSH-treated B16 cells. The data are representative of three independent experiments and expressed as the mean ± standard error of the mean (SEM). ^*∗*^*p* < 0.05, ^*∗∗*^*p* < 0.01, and ^*∗∗∗*^*p* < 0.001 compared with the untreated group (0 ppm) each at the same concentration.

To confirm whether CJS-EO contributed to the inhibition of melanin production, the difference in melanin secretion after treatment with each concentration of CJS-EO was analyzed under an environment promoting melanin production by *α*-MSH stimulation. CJS-EO was treated at concentrations of 31.25, 62.5, 125, 250, and 500 ppm, in the same manner as for tyrosinase activity. CJS-EO significantly inhibited melanogenesis in the *α*-MSH-treated group, and a significant amount of melanin was suppressed (Figure 3(b)). This was similar to the observation in the positive control, arbutin (Figure 3(a)). In particular, at the lowest concentration of 32.25 ppm in the treated group, the melanin content reduced significantly as compared with the untreated group (0 ppm). Therefore, it was concluded that CJS-EO effectively inhibited the synthesis or secretion of melanin in B16F10 cells. In the 31.25, 62.5, 125, 250, and 500 ppm CJS-EO-treated groups, the melanin content decreased by 1.1, 1.5, 2.6, 2.7, and 4.3 times, respectively, compared with the untreated group, indicating a prominent melanin secretion inhibitory effect. The cytotoxicity test, tyrosinase inhibition test, and melanin content test results indicate that CJS-EO is extremely valuable as a natural material in whitening cosmetics.

### 3.5. Genetic Investigation

**Figure 4:**
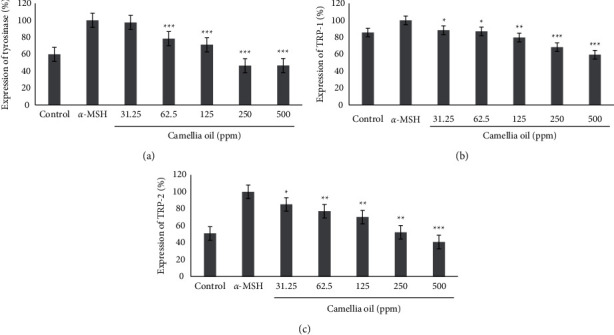
The effects of *C. japonica* seed essential oil (CJS-EO) on gene expression in *α*-MSH-treated B16 cells. (a) Expression of tyrosinase, (b) expression of TRP-1, and (c) expression of TRP-2. The data are representative of three independent experiments and expressed as the mean ± standard error of the mean (SEM). ^*∗∗∗*^*p* < 0.001 compared with *α*-MSH.

To determine the gene in CJS-EO that inhibits melanin biosynthesis by affecting the expression of genes involved in the melanin biosynthesis pathway, an experiment was performed using the RT-PCR method, and the results are shown in Figure 4. *α*-MSH was used as a negative control, and CJS-EO showed inhibitory effects on the expression of tyrosinase, TRP-1, and TRP-2 at all concentrations. In particular, CJS-EO inhibited the expression of tyrosinase by more than 50% at 125 ppm or higher. We discovered that the inhibitory efficacy of tyrosinase, TRP-1, and TRP-2 expression decreased as the concentration of CJS-EO increased. The results of this experiment suggest that the melanogenesis inhibitory effect of CJS-EO contributed to the inhibition of the expression of tyrosinase, TRP-1, and TRP-2.

### 3.6. Investigation of Protein Expression

**Figure 5:**
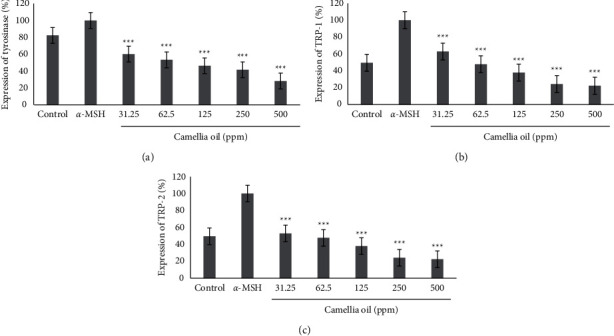
The effects of *C. japonica* seed essential oil (CJS-EO) on protein expression in *α*-MSH-treated B16 cells. (a) Expression of tyrosinase, (b) expression of TRP-1, and (c) expression of TRP-2. The data are representative of three independent experiments and expressed as the mean ± standard error of the mean (SEM). ^*∗*^*p* < 0.05, ^*∗∗*^*p* < 0.01, and ^*∗∗∗*^*p* < 0.001 compared to *α*-MSH.

The effect of CJS-EO on the expression of proteins involved in melanin biosynthesis was confirmed using western blotting, and the results are shown in Figure 5. When CJS-EO was compared with the *α*-MSH-treated group, which was a negative control group, it was confirmed that CJS-EO exhibited an inhibitory effect; in fact, CJS-EO exhibited the most prominent inhibitory effect on tyrosinase and TRP-2. The degree of inhibition was 46.6% and 40.7% at 500 ppm of tyrosinase and TRP-2, respectively, compared with the *α*-MSH-treated group. Although TRP-1 indicated the least inhibitory effect, it showed a significant decrease compared with the *α*-MSH-treated group. Therefore, the protein analysis showed the same trend as the gene analysis, and it was confirmed that CJS-EO stimulated tyrosinase, TRP-1, and TRP-2 to induce melanin reduction.

## 4. Discussion

Tyrosinase is an enzyme involved in melanin production via an enzymatic oxidative pathway, which determines the color of skin, hair, and eyes, as well as the browning of certain foods [29]. Chemical agents that demonstrate antityrosinase activity have been used in clinical medicine for the treatment of dermatologic disorders associated with melanin hyperpigmentation [30]. Melanin production may contribute to some of the histopathological features exclusive to malignant cancer [31]. Therefore, antityrosinase substances may facilitate the treatment of skin cancer. In recent years, the use of natural products instead of chemical or synthetic compounds has gained increasing interest as they are more economical, environmentally friendly, and safe [32]. Moreover, the research and development of green technology and low-cost raw materials are important to the industry as well as for improving the use of plant resources. Recently, the antityrosinase activity of certain plants has been reported [33]. However, data pertaining to the essential oils of natural plants are scarce. Therefore, the identification of plant essential oils that possess high antityrosinase activity has garnered significant interest. The etiology of pigmentation is not well understood. Melanin biosynthesis is catalyzed by melanocyte-specific enzymes such as TRP-1 and TRP-2 [34]. Tyrosinase is vital to melanocyte melanin biosynthesis and has been known for many decades [34]. Therefore, tyrosinase is an important index for the treatment of pigmentation. In our study, CJS-EO decreased melanin synthesis and affected antityrosinase activity; therefore, we concluded that the antimelanogenesis by CJS-EO may be associated with other enzymes (TRP-1 and TRP-2). Volatile substances have been reported to exhibit high antioxidant activities [35]. As volatiles, CJS-EO is characterized by a pungent odor. It is synthesized by plants as secondary metabolites and has been widely used owing to its bactericidal, virucidal, fungicidal, anticancer, antioxidant, and antidiabetic activities [36]. The CJS-EO investigated in this study was primarily composed of hexamethylcyclotrisiloxane, which constituted 42.36% of the total chemical content. *Toxicodendron vernicifluum* containing hexamethylcyclotrisiloxane has been shown to exhibit various biological and pharmacological activities, including central, antimicrobial, and antitumor activities [37]. In our study, high concentrations of CJS-EO indicated only slight cytotoxic effects on melanocytes. Therefore, the inhibitory effect of CJS-EO on *α*-MSH-induced B16F10 cell propagation was observed. The results showed that pretreatment with CJS-EO reduced *α*-MSH-induced cell propagation in a dose-dependent manner (Figure 3), and these results were similar with pure arbutin used as a positive control. In most natural essential oil used in traditional medicine, extracts containing complex compounds are used instead of pure compounds. These natural essential oils usually do not contain volatility compounds with highly specific bioactivities but volatility compounds with broader interactions [38]. Therefore, it is difficult for CJS-EO, which contains many components, to have a higher antimelanogenesis effect than pure arbutin. In the future, it is necessary to separate the volatile components contained in CJS-EO and study the antimelanogenesis effect on the separated single component. Based on our results, we tentatively conclude that CJS-EO can inhibit melanocytes from harmful factors such as TRP-1 (Figures 4 and 5). Therefore, we assumed that hexamethylcyclotrisiloxane was the major biologically active compound in CJS-EO. Existing studies regarding the whitening property of CJS-EO is insufficient; therefore, it can be used as basic data in future studies pertaining to main ingredients that exhibit whitening efficacy.

## 5. Conclusions

To the best of our knowledge, this is the first study to report the efficacy of CJS-EO in inhibiting melanin production in B16F10 melanoma cells. Our observations indicated that CJS-EO inhibited *α*-MSH-induced melanogenesis through tyrosinase inactivation and the simultaneous suppression of the expression of proteins involved in melanin biosynthesis in B16F10 melanoma cells. CJS-EO is known to be safe, and we confirmed that it is noncytotoxic in this study. Therefore, CJS-EO can potentially be employed as an effective skin-whitening agent for the future development of complementary and alternative medicine-based aromatherapy.

## Figures and Tables

**Table 1:** The gas chromatography-mass spectrometry (GC/MS) analysis of *C. japonica* seed essential oil (CJS-EO).

Compounds	Chemical formula	Molecular weight (g/mol)	Retention time (min)	Area (%)
Hexamethylcyclotrisiloxane	C_6_H_18_O_3_Si_3_	222.46	3.506	42.36
Octamethylcyclotetrasiloxane	C_8_H_24_O_4_Si_4_	296.62	8.378	23.28
Decamethylcyclopentasiloxane	C_10_H_30_O_5_Si_5_	370.77	12.850	5.81
5-Methyl-2-phenyl-1h-indole	C_15_H_13_N	207.27	13.919	0.56
2,6-Dimethoxy-phenol,	C_8_H_10_O_3_	154.16	15.750	0.31
Vanillin	C_8_H_8_O_3_	152.15	17.075	2.96
Cycloheptasiloxane	O_7_Si_7_	308.59	20.782	1.74
3,4-Dihydroxyphenylglycol	C_8_H_10_O_4_	170.16	24.079	1.10
2-(2-Methylpropyl)butanedinitrile	C_8_H_12_N_2_	136.19	26.935	0.43
n-Hexadecanoic acid	C_16_H_32_O	256.42	28.994	1.42
Cyclohexanone	C_18_H_19_NO_3_	297.3	29.479	0.35
Octadecanoic acid	C_18_H_36_O_2_	284.48	32.103	0.74
Hexanedioic acid	C_6_H_10_O	146.14	35.515	5.56
1,2-Benzenediol,3,5-bis(1,1-dimethylethyl)-	C_14_H_22_O_2_	222.32	35.943	0.49
Octasiloxane, octadecamethyl-	C_18_H_54_O_7_Si_8_	607.303	37.919	0.76
Formic acid	CH_2_O_2_	46.025	40.484	1.12
2,4,6-Cycloheptatrien-1-one	C_7_H_6_O	106.12	44.039	1.20
**Total**				**90.19**

## Data Availability

All datasets used and/or analyzed during the current study are available from the corresponding author on reasonable request.
